# Multiple Small Intestinal Neuroendocrine Tumors With Findings of Intestinal Obstruction

**DOI:** 10.7759/cureus.17629

**Published:** 2021-09-01

**Authors:** Mohammed H Basendowah, Mohammed A Ashour, Ammar Y Hassan, Shahad Alshaynawi, Lujain K Alyazidi

**Affiliations:** 1 Surgery, College of Medicine, King Abdulaziz University Hospital, Jeddah, SAU; 2 Medicine, College of Medicine, King Abdulaziz University Hospital, Jeddah, SAU; 3 Medical Physics, College of Medicine, King Abdulaziz University Hospital, Jeddah, SAU

**Keywords:** nets, intestinal obstruction due to sinets, sb-nets, si-nets, carcinoid tumors, small intestinal neuroendocrine tumors, gastric neuroendocrine tumors, neuroendocrine tumors, small bowl neuroendocrine tumors, net

## Abstract

Carcinoid tumors are one of the most common types of small intestinal neuroendocrine tumors (SI-NETs). However, SI-NETs that manifest as subacute intestinal obstruction are extremely rare. The annual occurrence rate of jejuno-ileal NETs is 0.28-0.8 per 100,000 people. In this report, we describe a case of subacute intestinal obstruction due to a mid-ileal stricture. The patient underwent laparotomy after evaluation and investigation. Mid-ileal growth was noted, and small bowel resection was performed with primary end-to-end anastomosis. Postoperative histopathology revealed the growth to be a well-differentiated NET.

## Introduction

Neuroendocrine tumors (NETs) are slow-growing, heterogeneous tumors that develop from neuroendocrine cells distributed all over the body. The lung, small intestine, rectum, and anus are the prominent organs affected by them [1]. Primary tumors of the small intestine are extremely rare, accounting for just 1-3% of all gastrointestinal (GI) tract cancers [2]. In fact, small intestinal neuroendocrine tumors (SI-NETs) have recently become more prevalent than adenocarcinoma. Consequently, SI-NETs have now become the most common GI neuroendocrine tumors [1]. In addition, the ileum and the jejunum are the most common sites for SI-NETs, accounting for 28% of all NETs [1,3]. From 1973 to 2004, the number of reported cases of NETs increased five-fold, from 1.09 to 5.25 per 100,000 people per year. Patients between the ages of 50 and 64 years have the highest prevalence of SI-NETs (38%), with males slightly more susceptible than females (5.35/100,000/year) [1].

Carcinoid tumors are the most common type of SI-NETs, even though they are a rare condition. Carcinoid tumors have an annual incidence of four to five cases per 100,000 individuals [4]. Usually, they are clinically silent, but they can cause pain, weight loss, abdominal mass, bowel obstruction, carcinoid syndrome, or perforation [5]. These tumors generally do not become large enough to induce intraluminal obstruction; instead, they cause obstruction by a local desmoplastic reaction [2].

We present a case of multiple SI-NETs with clinical and radiological findings of acute intestinal obstruction treated at King Abdulaziz University Hospital (KAUH), Jeddah.

## Case presentation

The patient was a 75-year-old male with a medical history of diabetes mellitus, hypertension, and benign prostatic hyperplasia. He presented to the Emergency Department (ED) complaining of recurrent, non-radiating paraumbilical colicky pain for six months. The pain was exacerbated by lying and relieved by sitting and bowel movements. It was associated with vomiting, abdominal distension, and constipation with alternating diarrhea. The patient had a history of hemorrhoidectomy 15 years ago. The abdominal examination was unremarkable, except for the presence of significant abdominal distention and mild umbilical tenderness.

**Figure 1:**
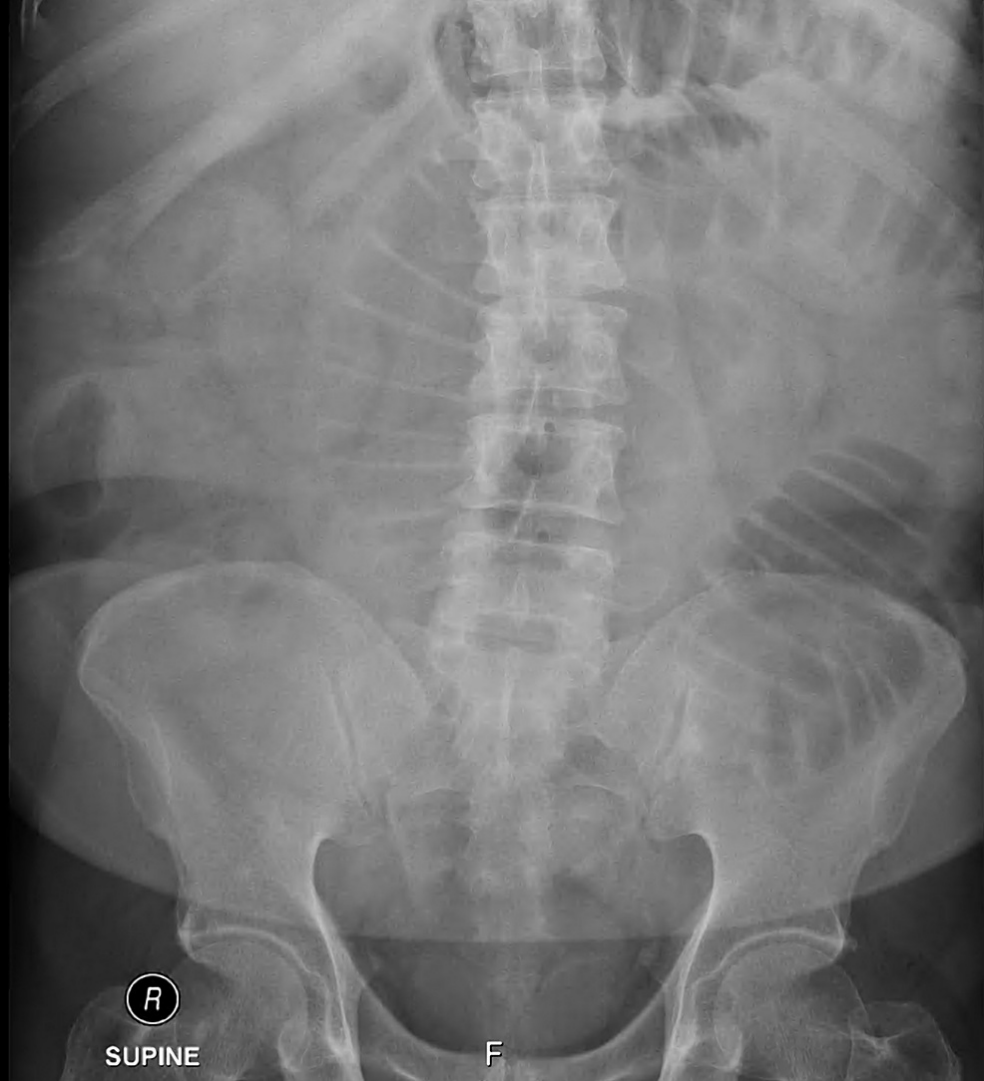
Plain abdominal radiograph showing multiple air-fluid levels indicating small bowel obstruction

**Figure 2:**
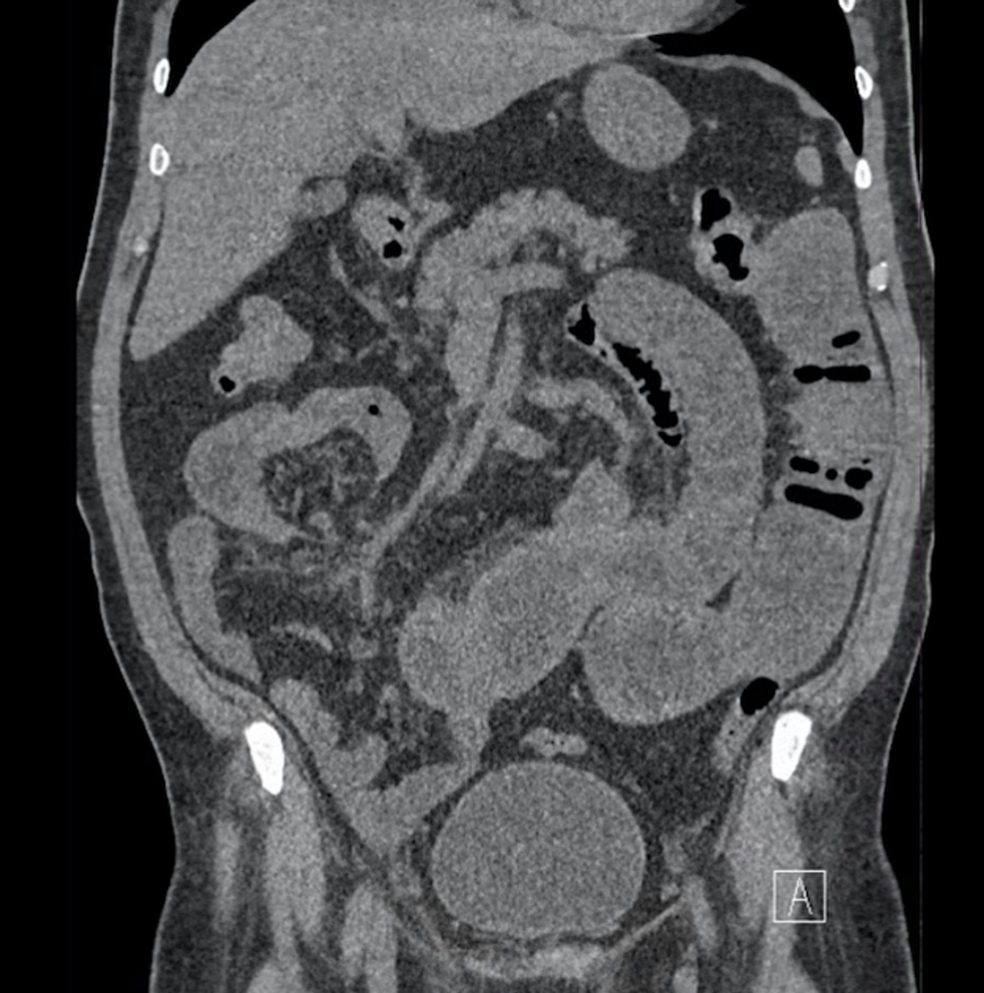
Coronal non-contrast CT KUB image CT: computed tomography; KUB: kidneys, ureters, and bladder

**Figure 3:**
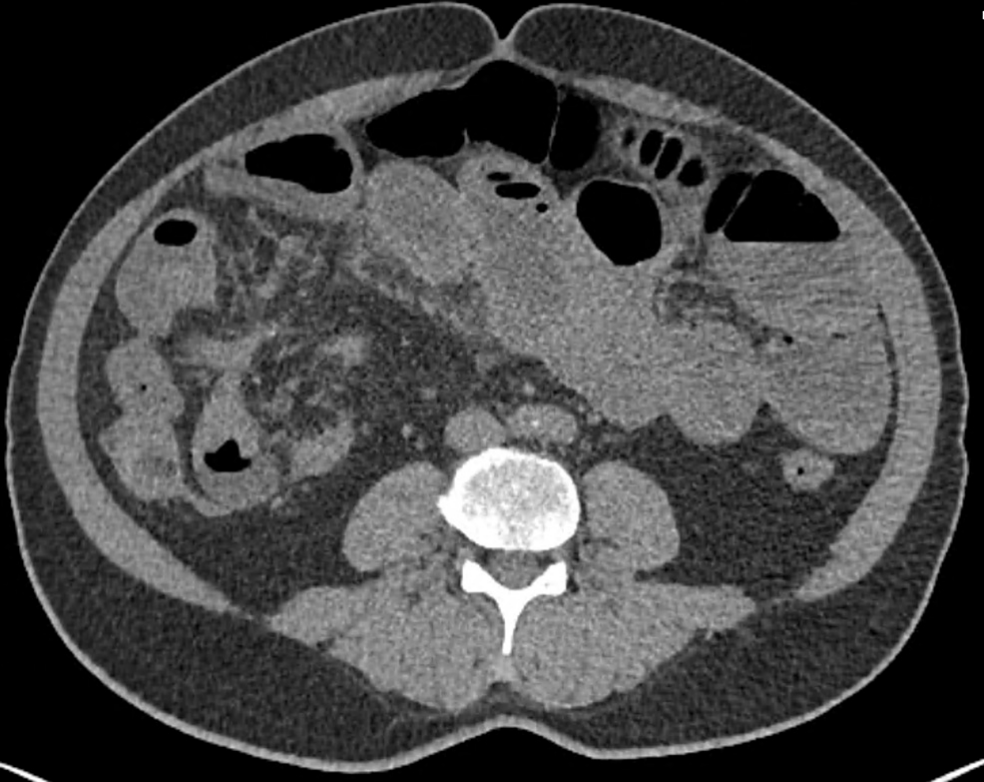
Axial non-contrast CT KUB image CT: computed tomography; KUB: kidneys, ureters, and bladder

An abdominal CT scan with IV contrast was done, which showed a dilatation in the small bowel reaching up to 4.8 centimeters with multiple air-fluid levels and transition zone at the distal ileal loops. There was circumferential mural thickening of the ileal loops in the right lower quadrant with surrounding fat strandings and prominent mesenteric lymph nodes. The rest of the ileal loops and the large bowel was collapsed. The constellation of findings was assumed to represent a neoplastic or inflammatory process. In addition, a plain abdominal radiograph was done, showing dilated small bowel loops, suggestive of small bowel obstruction (Figure 1). The CT scan images showed high-grade small bowel obstruction and transition zone with ileal wall thickening, fat stranding, and prominent lymph nodes (Figures 2, 3).

The patient underwent laparoscopic exploration, which revealed two mid ileal masses. The proximal part related to the masses was dilated, where the distal part was collapsed. The mid ileal masses were attached to the small bowel mesentery, and hence the procedure was converted to the midline laparotomy. The affected bowel was found 2.5 meters distal to the duodenojejunal (DJ) junction. Oncological resection of the masses was excluded to avoid superior mesenteric artery injury. Therefore, a small bowel resection accompanied by multiple intermediate size lymph node resection was made. At that time, side-to-side stapled anastomosis was also done. 

**Figure 4:**
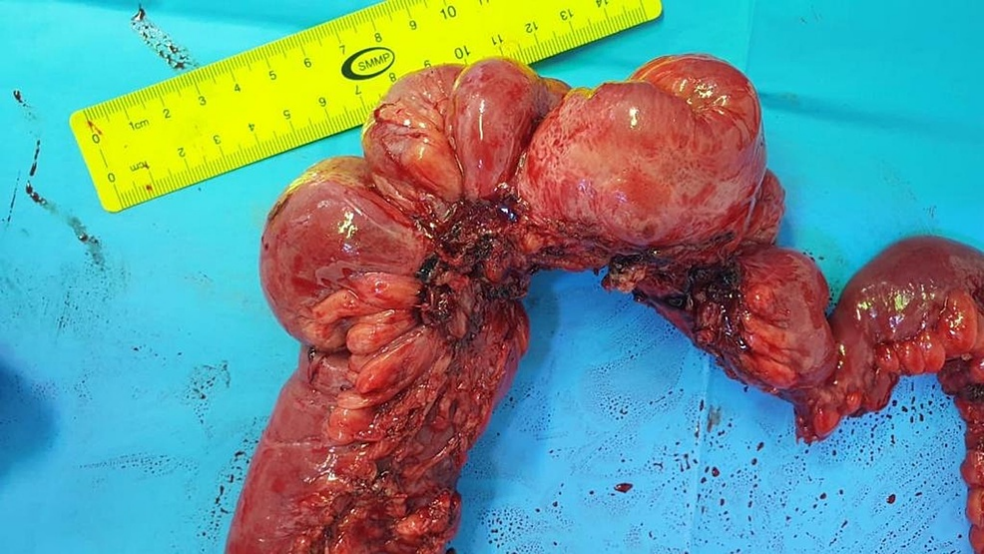
An intraoperative gross small bowel specimen shows dilated firm lumen due to the presence of palpable multiple tumors

Regarding histopathology, the measurement of the obstruction site was 11 x 5 cm. There were nine well-circumscribed, firm submucosal nodules, and the largest nodule measured 2.2 cm in its maximum dimension. The mesenteric lymph node specimens consisted of multiple fragments of fibrofatty tissue measuring 8 x 4 x 3 cm in aggregate (Figure 4). The histopathological findings showed grade 1 well-differentiated NETs of the mid-ileum with one metastatic lymph node (mpT2, N1). Furthermore, the muscularis propria was invaded by the tumors. Among the immune histochemistry markers, the tumors were positive for chromogranin A, synaptophysin, CD56, CDX2, and CKpan, while negative for CK7, TTF1, PAX8, CK20. 

The patient was discharged six days after the surgery in a stable and good condition. No adjuvant therapy was recommended since the tumors were well differentiated. He was discharged on enoxaparin, ciprofloxacin, and paracetamol. A follow-up after three months for physical examination, blood tests, and CT scan was recommended. After that, follow-ups should be done every 6-12 months for five years, and then every 12-24 months for 10 years.

## Discussion

Multiple tumors are seen in up to 33% of NETs cases. Therefore, it is crucial to inspect the whole length of the small bowel intraoperatively [4]. About 70% of SI-NETs are found in the ileum, making it the most common site [6]. Although the small intestine makes up 75% of the length of the GI tract and 90% of the absorptive mucosal surface area, tumors of the small intestine are rarer than other GI malignancies [1]. This could be attributed to the fact that the lumen of the small intestine is mainly free of bacteria. Thus, bacterial metabolites involved in genetic alterations are absent. In addition to the high levels of luminal immunoglobulin A (IgA), more significant distribution of lymphoid tissue in the small intestine epithelium, and high production of benzpyrene hydroxylase by enterocytes reduce the exposure of carcinogens [7]. The incidence of SI-NETs has significantly increased within the past decade, and this is due to the widespread use of imaging modalities and endoscopy in combination with the continuous update of the staging systems for neuroendocrine tumors globally [2,3].

Regarding its pathophysiology, SI-NETs are derived from Kulchitsky (enterochromaffin) cells, which are multipotent stem cells that migrate from the neural crest cells at the crypts of Lieberkuhn base to the gut ectoderm [2,8]. The obstruction of the intestine can be caused by peritumoral fibrosis or tumor invasion of the mesentery, which could cause kinking of the bowel, or secondary to desmoplastic reaction with scarring of the mass, which can cause ischemic effects [2,9]. The expression "carcinoid syndrome" refers to the hormonal symptoms of neuroendocrine tumors caused by the secretion of serotonin, bradykinins, and prostaglandins [2].

However, in primary tumors, the patient may be asymptomatic, and the tumor is slow-growing and relatively small; other manifestations include abdominal pain, diarrhea, weight loss, and bleeding. Moreover, intestinal obstruction caused by small bowel tumors is relatively rare. In fact, it may represent only 15-25 of the total cases [1,2]. It is crucial to note that around 6-30% of patients develop a carcinoid syndrome clinical presentation. In such cases, the presentations may manifest as chronic diarrhea, flushing attacks, bronchial constriction to severe pulmonary spasm, carcinoid heart disease with arrhythmias, hypo- or hypertension, and also abdominal pain or cramps [10]. In spite of this, in the absence of hepatic metastases, serotonin is degraded in the liver to non-active substances, preventing carcinoid syndrome manifestations [2]. Although our patient presented as a case of intestinal obstruction, there were no hormonal symptoms noticed due to the absence of hepatic metastasis.

Diagnosis of NETs in the early stages is challenging, and it may be accomplished through the grade of the mitotic count and Ki-67 index, which is considered the best marker for the cell proliferation state [1,11]. The World Health Organization (WHO)'s 2010 classification, supported by the European Neuroendocrine Tumor Society (ENETS), classifies NETs into three categories. Mitotic activity of less than 2 per 10 high power fields (HPFs) is seen in low-grade (G1) tumors with a mitotic index of less than 2%. Mitotic activity of 2-20 per HPF is found in intermediate grade (G2) tumors with a mitotic index of more than 3%, whereas a mitotic index of more than 20% or mitotic rate of more than 20 per HPF is found in grade 3 high-grade neuroendocrine carcinomas [2]. In our case, the mitotic counts of well-differentiated NETs that were presumed as low-grade or G1 were lower than 20 per 10 HPFs and the Ki-67 index level was less than 3%. Although radiological modality is rarely sufficient in the diagnosis of primary tumors, the clinical picture can be used to make a diagnosis, which can be assisted by imaging, endoscopy, or tumor markers such as chromogranin A and serotonin. CT is the ideal imaging modality when abdominal carcinoid tumors are suspected, as it generally reveals mucosal thickening, a submucosal mass, or luminal narrowing. Moreover, wall thickening and lymph node or hepatic metastasis can all be seen on ultrasound. In fact, video capsule endoscopy has been demonstrated to be more sensitive than standard CT and fluoroscopic barium investigations in detecting small intestine tumors for preoperative diagnosis and planning in patients with a suspected or known small intestinal tumors [2,4]. Despite the fact that the increased use of high-resolution imaging and endoscopy in clinical practice has led to a rise of early NET diagnosis, which supports a favorable prognosis, SI-NETs are still being discovered in a high percentage of cases after emergency surgery for acute bowel obstruction, perforation, and in rare cases of hemorrhages [10]. An example of this is our case, in which the patient underwent laparotomy exploration after he was admitted as a case of intestinal obstruction based on clinical and radiological findings.

According to multiple studies of research and observations, the most common site of metastasis for GI carcinoid tumors is the liver [4]. As reported in the literature, more than 50% of patients with NETs had distant metastasis at the prognosis stage. Approximately 7-80% of patients presented with liver and lymph node metastasis when they were diagnosed [12,13]. The size of the primary tumor has a direct impact on patient survival and metastasis rates. Tumors less than 1 cm are less likely to metastasize to the liver and have a 10% rate for lymph node metastasis, while tumors more than 2 cm in size showed a 28% rate of liver metastasis and a 72% rate for lymph node metastasis [4]. 

Regarding the treatment of SI-NETs, the stages play a crucial role. In stages I, II, and III, radical resection is performed to achieve the R0 stage. Besides, in the case of stage IV metastatic SI-NETs, the surgical approach must be preferred to achieve the least risk of local complications. In palliative settings, medical therapy is mandatory [1]. The goal of surgery is to gain symptomatic relief and improve survival rates even in patients with metastatic disease. A tumor size greater than 2 cm is usually associated with lymph node metastasis as well as a high risk of recurrence. Segmental resection is necessary with a wide broad clearance of the associated mesenteric lymph nodes. For tumor sizes less than 1 cm, local resection is satisfactory [2]. The standard treatment approach for SI-NETs is exploratory laparotomy with attentive palpation of the whole small bowel affected portion to explore any small and/or multifocal NETs [14]. For radical surgical resection, a thorough resection of the primary tumor, regional lymph nodes, and the associated mesenteric fibrosis should be involved, followed by an end-to-end anastomosis of the bowel [10,15]. Patients with hormonal symptoms can be managed with biotherapy using drugs such as somatostatin analogs; 70-80% of these patients showed symptomatic improvement as well as stabilization for tumor growth. It is used as a first-line treatment in inoperable functioning tumors and to avoid the risk of carcinoid crisis in candidates for surgery. Regarding the future management of NETs, sensitive biomarkers are expected to be developed adjacent to molecular imaging, which would direct the treatment based on tumor biology and molecular genetics. They would provide new therapeutic approaches, based on the distinctive features of NETs [3].

The prognosis of SI-NETs depends on both stage and grade. According to the WHO classification of 2010, the five-year survival rate for stages I and II is 100%, while that for stage III is 97.1%, and for stage IV, it is 84.8%. According to grading, patients with G1 SI-NETs have a 93.8% five-year survival rate, while those with G2 have 83.0%, and patients with the relatively rare G3 have only about 50% [1]. In fact, when hepatic metastases are present, the five-year survival rate for both jejunal and ileal tumors is as low as 18% [3]. The characteristics of aggressive tumors that will require a surgical approach include tumor size larger than 1 cm, presence of metastasis to the locoregional lymph node, or mesenteric spread [9]. Actually, the survival is higher among patients aged less than 50 years at the time of diagnosis, those with tumor sizes of less than 2 cm, those with tumors in the duodenal location, and those with TNM T or T2 with N0 as well as those who undergo complete surgical resection [1]. Additionally, proliferative activity, which includes mitotic activity and the Ki67 proliferation index, has been discovered to be a good prognostic predictor [2]. In our case, the patient was more than 50 years in age, suffering from multiple mid-ileal masses with the largest tumor of 2.2 cm. The pathological stage classification of our patient was found to be mpT2, N1 after a complete small bowel surgical resection was made.

## Conclusions

We presented the case of a patient with SI-NETs, particularly in the mid-ilium, which manifested as intestinal obstruction. Our case highlights the importance of keeping a high index of suspicion for NETs in patients with small bowel obstruction, especially if the risk factors we mentioned above are present. Finally, the treatment approach and management that were employed in this case represent the optimum approach toward any case where SI-NETs are suspected.
